# Genomic Profiling of Messenger RNAs and MicroRNAs Reveals Potential Mechanisms of TWEAK-Induced Skeletal Muscle Wasting in Mice

**DOI:** 10.1371/journal.pone.0008760

**Published:** 2010-01-19

**Authors:** Siva K. Panguluri, Shephali Bhatnagar, Akhilesh Kumar, John J. McCarthy, Apurva K. Srivastava, Nigel G. Cooper, Robert F. Lundy, Ashok Kumar

**Affiliations:** 1 Department of Anatomical Sciences and Neurobiology, University of Louisville School of Medicine, Louisville, Kentucky, United States of America; 2 Department of Physiology, College of Medicine, University of Kentucky, Lexington, Kentucky, United States of America; 3 Laboratory of Human Toxicology and Pharmacology, Applied & Developmental Research Directorate SAIC-Frederick, National Cancer Institute, Frederick, Maryland, United States of America; University Hospital Vall d'Hebron, Spain

## Abstract

**Background:**

Skeletal muscle wasting is a devastating complication of several physiological and pathophysiological conditions. Inflammatory cytokines play an important role in the loss of skeletal muscle mass in various chronic diseases. We have recently reported that proinflammatory cytokine TWEAK is a major muscle-wasting cytokine. Emerging evidence suggests that gene expression is regulated not only at transcriptional level but also at post-transcriptional level through the expression of specific non-coding microRNAs (miRs) which can affect the stability and/or translation of target mRNA. However, the role of miRs in skeletal muscle wasting is unknown.

**Methodology/Principal Findings:**

To understand the mechanism of action of TWEAK in skeletal muscle, we performed mRNA and miRs expression profile of control and TWEAK-treated myotubes. TWEAK increased the expression of a number of genes involved in inflammatory response and fibrosis and reduced the expression of few cytoskeletal gene (e.g. Myh4, Ankrd2, and TCap) and metabolic enzymes (e.g. Pgam2). Low density miR array demonstrated that TWEAK inhibits the expression of several miRs including muscle-specific miR-1-1, miR-1-2, miR-133a, miR-133b and miR-206. The expression of a few miRs including miR-146a and miR-455 was found to be significantly increased in response to TWEAK treatment. Ingenuity pathway analysis showed that several genes affected by TWEAK are known/putative targets of miRs. Our cDNA microarray data are consistent with miRs profiling. The levels of specific mRNAs and miRs were also found to be similarly regulated in atrophying skeletal muscle of transgenic mice (Tg) mice expressing TWEAK.

**Conclusions/Significance:**

Our results suggest that TWEAK affects the expression of several genes and microRNAs involved in inflammatory response, fibrosis, extracellular matrix remodeling, and proteolytic degradation which might be responsible for TWEAK-induced skeletal muscle loss.

## Introduction

Skeletal muscle wasting or atrophy is a major cause of human morbidity [1], [2], [3]. Proinflammatory cytokines are the key mediators of muscle-wasting in various chronic conditions [4], [5]. Besides directly inducing the degradation of selective muscle proteins [6], [7], elevated levels of inflammatory cytokines cause extracellular matrix abnormalities [8] and prevents the regeneration of skeletal muscle fibers by inhibiting the differentiation of muscle progenitor cells into myofibers [9], [10]. Accumulating evidence suggests that bulk of the muscle protein degradation in atrophying skeletal muscle occurs through the activation of ubiquitin-proteasome system [4], [11], [12]. In addition, it has been also found that muscle-wasting conditions involve the activation of nuclear-factor-kappa B (NF-κB), a proinflammatory transcription factor, which regulates the expression of large number of genes including the components of ubiquitin-proteasome system [1], [13]. Specific inhibition of NF-κB activity has been found to attenuate loss of skeletal muscle mass in response to various catabolic stimuli including proinflammatory cytokines, tumor load, denervation, and unloading [13], [14], [15], [16].

TNF-like weak inducer of apoptosis (TWEAK) is an important inflammation-related cytokine belonging to TNF super family ligands [17], [18]. The actions of TWEAK in target cells are mediated through its binding to Fn14, a type I transmembrane receptor, belonging to the TNF receptor super family [17], [19]. Recently, we have reported that treatment of myotubes with TWEAK leads to the degradation of select muscle proteins, which in turn leads to atrophy, thus signifying TWEAK as a major muscle-wasting cytokine [20]. In fact, we have found that at equimolar concentrations, TWEAK is more potent than its structural homologue and well known muscle-wasting cytokine TNF-α to induce the degradation of myosin heavy chain (MyHC) in cultured myotubes [20]. Chronic administration of soluble TWEAK protein or transgenic overexpression of TWEAK in mice also causes significant muscle-wasting [20]. TWEAK also inhibits the differentiation of myoblasts into multinucleated myotubes and induces the degradation of myogenic regulatory factors (MRFs) such as MyoD [21], [22]. Our recent studies have further suggested that the expression of TWEAK receptor Fn14 is increased in skeletal muscle in disuse conditions (e.g. immobilization, unloading, and denervation) and TWEAK is the major mediator of skeletal muscle loss in response to denervation (Mittal et al., unpublished observations). However, the underpinning mechanisms by which TWEAK induces skeletal muscle loss remain largely unknown.

Previous examinations of genome-wide gene expressions in skeletal muscle has helped in identifying several known and novel genes which mediate the loss of skeletal muscle mass in disuse conditions such as unloading, sarcopenia, starvation, and denervation [23], [24], [25], [26], [27]. However, the effects of proinflammatory cytokines such as TWEAK on the gene expression and intracellular pathways related to the acquisition and maintenance of skeletal muscle mass remain unknown. MicroRNAs (miRNAs or miRs), a new class of non-translating RNAs, plays critical role as molecular switches for complex and extensive regulatory web involving thousands of genes [28], [29]. MicroRNAs are small 18 to 22 nucleotide long RNA molecules, which negatively regulate expression of target genes by binding to specific sequences in 3′UTR where partial complementarities inhibit their translation and perfect complementarily induces degradation of mRNA [28], [29]. With the advent of these tiny regulatory RNAs, the complexity of understanding the regulatory mechanisms of many important pathways has been resolved [28], [29], [30]. miRs have been shown to regulate a range of biological processes including tumorigenesis, development of the limb, lung and hematopoietic systems, and adipogenesis [30]. Furthermore, a few skeletal muscle specific miRs (e.g. miR-1, miR-133, and miR-206) have been characterized as modulators of myogenic cells proliferation and differentiation [31], [32], [33] and there is increasing evidence about the involvement of miRs in skeletal muscle disorders such as muscular dystrophy [34], [35], [36]. However, the role of miRs and their potential gene targets in atrophying skeletal muscle remain completely unknown. Identification and understanding the mechanisms of actions of miRs that are differentially regulated in atrophying skeletal muscle may provide novel molecular targets towards therapeutic approaches in muscle-wasting.

In this study, using cDNA microarray, low density microRNA array, TaqMan PCR assays, and bioinformatics tools, we have investigated the potential mechanisms by which TWEAK regulates skeletal muscle mass. Our results suggest that TWEAK modulates the expression of selective muscle genes and miRs in cultured myotubes and in skeletal muscle-specific TWEAK-Tg mice. Furthermore, bioinformatics analyses of differentially regulated genes and miRs have shown that TWEAK affects diverse cellular responses such as proliferation, musculature development, inflammation, and adipocyte formation.

## Results

We have previously shown that treatment of C2C12 myotubes with TWEAK augments the expression of muscle-specific E3 ubiquitin ligases atrogin and MuRF1 and augments the ubiquitination of select muscle proteins within 12–24h of treatment [20]. In this study, we have performed mRNA and miRNA profiling after 18h of TWEAK treatment to detect the expression of both early and late responsive genes. To validate the effects of TWEAK on expression of various genes and miRs *in vivo*, we have also employed TWEAK-Tg mice. We have previously reported that transgenic mice expressing very high levels (>14 fold) of TWEAK in skeletal muscle died at perinatal/neonatal age [20]. However, the mice which expressed relatively low levels of TWEAK (4–5 folds higher than littermate controls) survived and developed into adulthood. Our recent analysis of skeletal muscle revealed that TWEAK-Tg mice show significant muscle fiber atrophy at the age of 4–6 months (Mittal et al (2009), unpublished observation). Therefore, we used skeletal muscle from 6-months old TWEAK-Tg and their littermate control mice.

### Microarray Analysis of Global Gene Expression in TWEAK-Treated C2C12 Myotubes

**Figure 1:**
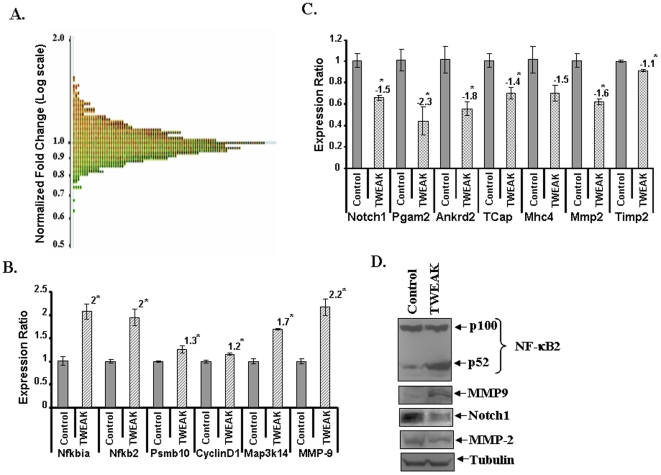
Differential expression of genes by TWEAK in C2C12 myotubes. **A).** Distribution curve of differentially expressed genes in response to TWEAK treatment detected by cDNA microarray analysis. The normalized fold changes were plotted on y-axis on logarithmic scale. **B & C).** C2C12 myotubes were treated with 10 ng/ml of TWEAK for 18h followed by isolation of total RNA and QRT-PCR. Untreated cells under similar conditions were taken as control. The relative expression values from the QRT-PCR analysis were plotted for each gene are mean ± SD (n = 3). The numbers above the bar represents the fold changes with TWEAK treatment against control, and ‘*’ represents the statistical significance (p-value ≤0.01). Data presented here show that mRNA levels of Nfkbia, Nfkb2, cyclinD1, Map3k14, and Mmp9 was significantly increased whereas the levels of Notch1, Pgam2, Ankrd2, TCap, Mhc4, Mmp2, and Timp2 are reduced in TWEAK-treated C2C12 cells. The relative expression values from the QRT-PCR analysis were plotted for each gene are mean ± SD (n = 3). The numbers above the bar represents the fold changes with TWEAK treatment against control, and ‘*’ represents the statistical significance (p-value ≤0.01). **D).** Differential expression of NF-κB2, MMP-9, Notch1, and MMP-2. C2C12 myotubes were treated with 10 ng/ml of TWEAK for 18h following isolation of total protein for Western blotting. All the samples were quantified and equal amounts of proteins were loaded on 10% SDS-PAGE gel. Representative immunoblots from three independent experiments (n = 3) presented here showed that TWEAK treatment increases the protein levels of NF-κB2 and MMP-9 and reduces the levels of Notch1 and MMP-2.

C2C12 myotubes were treated with TWEAK (10ng/ml) and the mRNA level of different genes was monitored by cDNA microarray technique. The microarray gene expression profile appeared normally distributed for TWEAK-treated samples (Figure 1A) indicating that our analysis of differentially expressed gene is not biased due to skewed distribution of certain genes. Out of approximately 25,000 genes present on our microarray chips, TWEAK significantly (p<0.05) affected the expression of a total of 6,938 genes (2,841 up regulated and 4,097 down regulated). Top 50 up-regulated and top 50 down-regulated known genes in TWEAK-treated myotubes are presented in Table S1. Further analysis of differentially regulated genes showed that about 67 genes were up-regulated and 26 down-regulated with fold values ≥1.5 and p-value of ≤0.05. Only 12 up-regulated genes (e.g. Nfkbia, Taf2 and Slc2a6 etc.) were with fold values ≥2. We further observed that 13 out of 26 significantly down-regulated genes were less than 2-fold and the functions of 10 genes with fold value more than two is not yet known (Table. 1). Row and normalized data of this microarray experiment has been submitted to ArrayExpress database (http://www.ebi.ac.uk/microarray-as/ae/) with accession number E-MEXP-2432.

**Table 1:** List of differentially expressed genes in TWEAK-treated C2C12 cells by cDNA microarray with p-values ≤0.05 and fold ≥1.5.

Gene Name	p-value	Fold	Gene Description
mCA038616	0.000272	−2.90698	
mCD037457	4.54E-06	−2.7933	
mCD037931	1.89E-05	−2.6455	
mCD037944	0.00676	−2.457	
mCD037194	0.000753	−2.3753	
mCD037554	0.00103	−2.3753	
mCD037733	0.0163	−2.33645	
mCD037751	0.000229	−2.331	
mCD037542	0.0103	−2.25225	
mCD037061	0.000161	−2.21239	
mCD037073	0.000524	−1.98413	
mCD037555	0.00532	−1.8315	
mCD036875	0.0237	−1.80505	
Ankrd2	0.000605	−1.74825	ankyrin repeat domain 2 (stretch responsive muscle)
Pgam2	0.00018	−1.66113	phosphoglycerate mutase 2
mCA037854	0.000655	−1.63934	Bacillus subtillis sporulation protein (spoOB), GTP-binding protein (obg), phenylalanine biosynthesis associated protein (pheB), and monofunctional prephenate dehydratase (pheA) genes, complete cds.
Myh4	2.71E-05	−1.63666	myosin, heavy polypeptide 4, skeletal muscle
Prelp	5.54E-05	−1.61812	proline arginine-rich end leucine-rich repeat
1110059G02Rik	5.11E-05	−1.60772	RIKEN cDNA 1110059G02 gene
Notch1	0.0192	−1.5949	Notch gene homolog 1 (Drosophila)
4732473B16Rik	0.0496	−1.5949	RIKEN cDNA 4732473B16 gene
Tcap	1.03E-06	−1.57978	titin-cap
Idb3	8.94E-05	−1.55039	inhibitor of DNA binding 3
Depdc6	5.29E-05	−1.53846	DEP domain containing 6
Olfr297	0.00123	−1.51745	olfactory receptor 297
Nrap	0.00014	−1.51515	nebulin-related anchoring protein
Isyna1	0.0314	1.501	myo-inositol 1-phosphate synthase A1
Fkhl18	0.00199	1.502	forkhead-like 18 (Drosophila)
Map3k14	0.00252	1.503	mitogen-activated protein kinase kinase kinase 14
1110020C13Rik	0.00967	1.503	RIKEN cDNA 1110020C13 gene
Mmp9	0.00172	1.509	matrix metalloproteinase 9
mCD037530	5.17E-05	1.521	
C030034P18Rik	0.0127	1.525	RIKEN cDNA C030034P18 gene
V1rd21	0.000812	1.526	vomeronasal 1 receptor, D21
mCD037434	0.00167	1.53	
2310031L18Rik	0.0193	1.53	RIKEN cDNA 2310031L18 gene
Cd200r4	0.00119	1.531	Cd200 receptor 4
Psmb10	0.000434	1.536	proteasome (prosome, macropain) subunit, beta type 10
Olfr1392	0.0131	1.544	olfactory receptor 1392
Nmyc1	0.00181	1.545	neuroblastoma myc-related oncogene 1
Lxn	5.39E-05	1.547	latexin
Pnn	0.0054	1.549	pinin
Zfp9	0.00695	1.553	zinc finger protein 9
AF310134	0.00289	1.568	Mus musculus krev interaction trapped 1 mRNA, complete cds.
C730014E05Rik	0.0467	1.569	RIKEN cDNA C730014E05 gene
mCT038085	0.00587	1.575	
Olfr186	0.000217	1.577	olfactory receptor 186
Krt1-14	0.000641	1.579	keratin complex 1, acidic, gene 14
Cstb	0.000164	1.581	cystatin B
9430078K10Rik	0.0288	1.582	RIKEN cDNA 9430078K10 gene
V1rd18	4.72E-05	1.602	vomeronasal 1 receptor, D18
Defb1	0.00235	1.602	defensin beta 1
Hsd17b9	0.00192	1.605	hydroxysteroid (17-beta) dehydrogenase 9
AI182371	1.01E-05	1.606	expressed sequence AI182371
B230208H21	0.00961	1.606	hypothetical protein B230208H21
BC051076	0.000235	1.607	cDNA sequence BC051076
1200014M14Rik	0.00663	1.607	RIKEN cDNA 1200014M14 gene
Gzmb	0.000451	1.608	granzyme B
Slc9a3	0.00148	1.609	solute carrier family 9 (sodium/hydrogen exchanger), member 3
4933433J03Rik	0.00449	1.634	RIKEN cDNA 4933433J03 gene
mKIAA1696	0.00206	1.638	Mus musculus mRNA for mKIAA1696 protein.
mCD037717	0.0475	1.638	
Adam2	0.00157	1.644	a disintegrin and metalloprotease domain 2
mCD037318	0.0491	1.663	
Krtap16-2	9.19E-05	1.673	keratin associated protein 16-2
H2-K1	0.00429	1.681	histocompatibility 2, K1, K region
Mt2	0.000112	1.701	metallothionein 2
C3	0.000787	1.719	complement component 3
mCD037656	0.0268	1.729	
5730530J16Rik	0.000173	1.737	RIKEN cDNA 5730530J16 gene
X66118	0.000695	1.742	M.musculus mRNA for glutamate receptor subunit GluR5-2c.
Nfkb2	0.000273	1.762	nuclear factor of kappa light polypeptide gene enhancer in B-cells 2, p49/p100
mCD037577	0.00367	1.781	
4930432K09Rik	0.000919	1.792	RIKEN cDNA 4930432K09 gene
Polr3k	0.0222	1.798	polymerase (RNA) III (DNA directed) polypeptide K
mCA038549	0.000136	1.803	
4930580F03Rik	0.000366	1.821	RIKEN cDNA 4930580F03 gene
mCD037266	0.00368	1.852	
2300002C06Rik	6.83E-05	1.859	RIKEN cDNA 2300002C06 gene
mCD037925	0.0191	1.865	
Dlgap2	0.0379	1.865	discs, large (Drosophila) homolog-associated protein 2
mCD037088	0.00288	1.971	
mCN038213	0.0004	1.991	
Slc2a6	3.20E-05	2.142	solute carrier family 2 (facilitated glucose transporter), member 6
mCD037835	0.000599	2.17	
mCD037271	9.84E-06	2.171	
E230016D10	0.00575	2.183	hypothetical protein E230016D10
mCD037850	0.000558	2.204	
mCD037361	0.00522	2.37	
Nfkbia	2.16E-05	2.54	nuclear factor of kappa light chain gene enhancer in B-cells inhibitor, alpha
mCA038179	0.026	2.709	
Taf2	0.00065	2.761	TAF2 RNA polymerase II, TATA box binding protein (TBP)-associated factor, 150kDa
mCD037476	2.60E-07	3.788	

**Figure 2:**
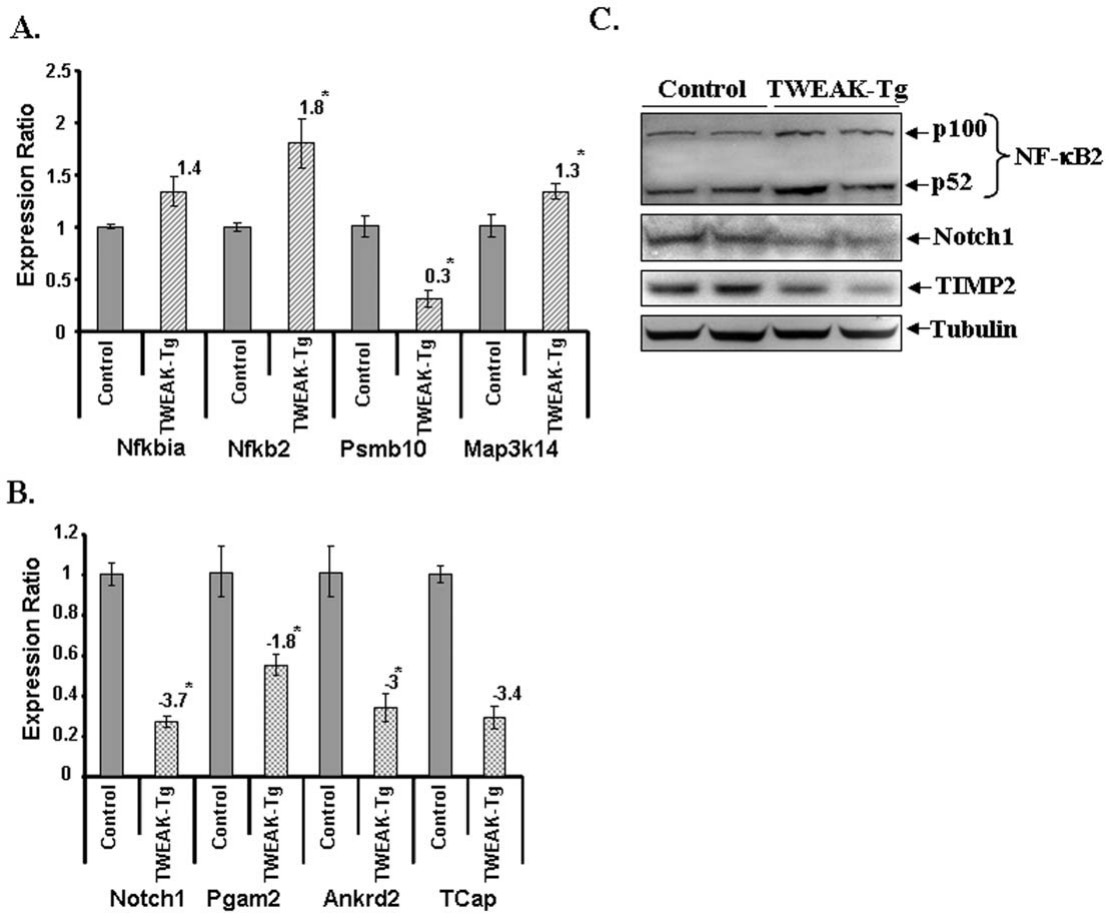
Differential expression of genes in skeletal muscle of TWEAK-Tg mice. Gastrocnemius muscle of 6 months old TWEAK-Tg mice and littermate control mice were used for total RNA isolation and QRT-PCR analysis. The relative expression values from the QRT-PCR analysis were plotted for each gene are mean ± SD (n = 3). The numbers above the bar represents the fold changes in TWEAK-Tg against littermate control mice, and ‘*’ represents the statistical significance (p-value ≤0.01). **A)** The levels of Nfkbia, Nfkb2, and Map3k14 were increased whereas the level of Psmb10 was found to be reduced in TWEAK-Tg mice compared to littermate control mice (n = 3 in each group). **B).** QRT-PCR analysis showed that the levels of Notch1, Pgam2, Ankrd1, and TCap were reduced in TWEAK-Tg mice compared to control mice (n = 3 in each group). **C).** Western blot analysis of NF-κB2, Notch1 and TIMP2 protein expression profiles in TWEAK-Tg compared to control mice. The gel pictures presented here from two independent experiments (n = 4) showed that protein levels of Notch1, and TIMP-2 were significantly reduced whereas NF-κB2 protein levels were increased in gastrocnemius muscle of TWEAK-Tg compared to littermate control.

Independent QRT-PCR assays were performed for the genes which showed high fold change and/or have a direct or indirect relation with skeletal muscle wasting. As shown in Figure 1B, the expression of Nfkbia, Nfkb2, Psmb10, cyclin D1, Map3k14, and Mmp9 was found to be significantly increased in TWEAK-treated samples in QRT-PCR assays. Similarly, the reduced expression of Notch1, Pgam2, Ankrd2, TCap, MyHC4, MMP-2 and TIMP2 in TWEAK-treated samples was confirmed by independent QRT-PCR assays (Figure 1C) suggesting direct correlation between microarray and QRT-PCR analysis for almost all the genes tested. Consistent with their mRNA levels, the protein levels of NF-κB2, and MMP-9 were also increased whereas the levels of Notch1 and MMP-2 were reduced in TWEAK-treated myotubes determined by Western Blot (Figure 1D). QRT-PCR analysis further showed that the expression levels of Nfkbia, Nfkb2, and Map3k14 were significantly up-regulated in skeletal muscle of TWEAK-Tg mice (Figure 2A). However, in contrast to TWEAK-treated myotubes, the expression of Psmb1 was found to be significantly reduced in skeletal muscle of TWEAK-Tg mice compared to littermate control mice (Figure 2A). Although the exact reasons for this anomalous regulation of Psmb1 in cultured myotubes and TWEAK-Tg mice is not yet clear, it is possible that continued presence of TWEAK in skeletal muscle of transgenic animal may lead to its reduced expression due to compensatory (negative feed-back) mechanisms. On the other hand, the reduced mRNA levels of Notch1, phosphoglycerate mutase 2 (PGAM2), ankyrin repeat domain 2 (Ankrd2), and TCap in skeletal muscle of TWEAK-Tg mice (Figure 2B) was consistent with the data obtained in TWEAK-treated C2C12 myotubes. Consistent with cell culture data, the protein level of NF-κB2 was increased whereas the levels of Notch1 and TIMP-2 were diminished in gastrocnemius muscle of TWEAK-Tg mice compared to control mice (Figure 2C). Collectively, these data indicate that our microarray analysis represents the set of the genes that are differentially regulated in response to TWEAK.

### TWEAK Regulates the Activity of Toxic Pathways in Myotubes

To understand the effects of TWEAK on various canonical pathways, we used Ingenuity Pathway Analysis (IPA) software. We first used a set of differentially regulated genes with fold values ≥1.5 and p-value of ≤0.05 in microarray analysis as an input in IPA software. However, this set of genes was not sufficient to generate pathways affected by TWEAK. We then reduced the stringency and used the set of genes with fold change (both up- and down-regulated genes) values ≥1.2 and p-value of ≤0.05 in the microarray experiment. We found that TWEAK affects the expression of genes that are involved in distinct molecular pathways. The major pathways affected by TWEAK in myotubes were those that regulate hepatic fibrosis, oxidative stress, NF-κB, mitochondrial dysfunction, TGF-β, and anti-apoptotic response (Table 2). Interestingly, our bioinformatics analysis of pathways using differentially regulated gene is consistent with the experimental evidence that skeletal muscle-wasting and other muscular disorders such as muscular dystrophy involves the activation of some/all of these molecular pathways [1], [2], [4], [37], [38], [39]. These data suggest that TWEAK may utilize many common pathways that are also activated by other catabolic stimuli to cause the loss of skeletal muscle mass and accumulation of fibrotic tissues (Table 2).

**Table 2:** List of top 20 toxicity pathways induced by TWEAK in C2C12 cells. Ingenuity pathway analysis was used to generate the toxicity pathways involved by differentially expressed genes by TWEAK with p-values≤0.05 and ≥1.2-fold. Negative logarithmic p-values in the table are Fisher's exact test p-value which determines the probability of the association between the genes in the data set and the canonical pathway. Ratio was calculated by the genes in the data set involved in a particular toxicity pathway divided by total number of genes involved in that pathway.

Ingenuity Toxicity Lists	−Log(P-value)	Ratio	Molecules
Hepatic Fibrosis	5.640	0.200	ELN, IGFBP6, LEP, BGN, COL4A3, MMP2, IGFBP7, COL4A2, COL1A2, COL5A1, CCL2, CSF1, TIMP1, TGFB3, SPARC, MMP9, AGT
Hepatic Stellate Cell Activation	3.360	0.229	RELA, CCL2, TIMP1, TGFB3, NFKB2, NFKB1, PDGFRB, AGT
RAR Activation	2.280	0.113	ADCY9, RELA, STAT5A, ADCY3, MAPK13, NFKB2, NFKB1, RXRG, AKT1, JUN, TAF4, DUSP1, CRABP2, TGFB3, PRKCB
NFκB Signaling Pathway	1.410	0.098	MAP3K14, RELA, AKT1, NFKBIA, BCL10, HDAC1, TNFAIP3, MAP3K8, TRAF5, NFKB2, NFKB1
G1/S Transition of the Cell Cycle	1.370	0.125	RB1, CCNE2, HDAC1, TGFB3, CCND1, SKP1
Oxidative Stress Response Mediated by Nrf2	1.060	0.078	GSTA3, NQO1, MAF, SLC35A2, DNAJC13, DNAJC3, CLPP, TXNRD1, GSR, AKT1, JUN, ERP29, KEAP1, ACTA1, EPHX1, PRKCB
TGF-β Signaling	0.920	0.091	JUN, GRB2, HDAC1, TGFB3, ACVR2B, SMAD5, SERPINE1
Mitochondrial Dysfunction	0.864	0.080	GSR, NDUFB7, UQCRC2, CYB5R3, COX3, CYCS (includes EG:54205), OGDH, UQCRC1, COX7A1, TXNRD2
Cholesterol Biosynthesis	0.674	0.125	ACAT1, HMGCR
Anti-Apoptosis	0.608	0.094	HDAC1, TNFAIP3, BCL2L10
G2/M Transition of the Cell Cycle	0.558	0.088	WEE1, BRCA1, SKP1
Hepatic Cholestasis	0.518	0.067	ADCY9, MAP3K14, RELA, JUN, NFKBIA, ADCY3, NFKB2, NFKB1, PRKCB
LPS/IL-1 Mediated Inhibition of RXR Function	0.512	0.064	GSTA3, SLC27A5, LIPC, JUN, CHST7, FABP4, SLC35A2, CHST12, CES2 (includes EG:8824), FABP3, ALDH3A1, ALDH9A1
Hypoxia-Inducible Factor Signaling	0.488	0.071	AKT1, JUN, NQO1, ELAVL1, PRKCB
Cytochrome P450 Panel - Substrate is a Fatty Acid (Human)	0.371	0.100	PTGIS
CAR/RXR Activation	0.330	0.069	GSTA3, CCND1
Positive Acute Phase Response Proteins	0.284	0.063	C3, SERPINE1
Fatty Acid Metabolism	0.262	0.054	SLC27A5, ACAA1, ACAT1, ALDH3A1, ALDH9A1, GCDH, ADH4

### Identification of Differentially Expressed MicroRNAs (miRs) in TWEAK-Treated Myotubes

**Figure 3:**
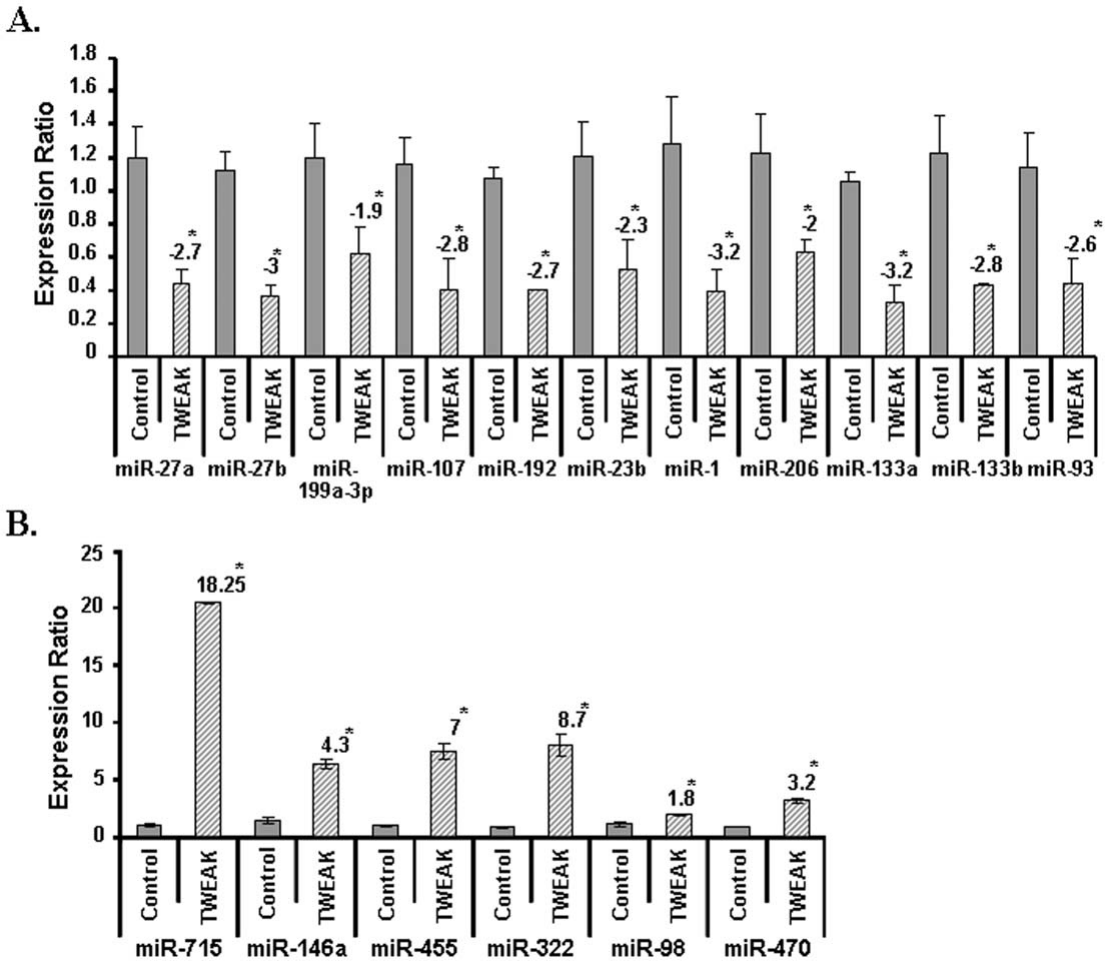
Differential expression of miRNAs in TWEAK-treated C2C12 myotubes measured by low-density miRNA array. **A)** C2C12 myotubes were treated with 10ng/ml of TWEAK for 18h following isolation of total RNA enriched with small RNAs. Untreated C2C12 myotubes under exactly similar conditions served as control. The normalized expression ratios were plotted for each miRNA are mean ± SD (n = 3). Low-density miRNA array of TWEAK-treated C2C12 myotubes showed down-regulation of miR-1, miR-133a, miR-133b, miR-206, miR-27, miR-23, miR-93, miR-199, miR-107, and miR-192. The numbers above the bar represents the fold changes with TWEAK treatment against control with p-values ≤0.05. **B).** TWEAK increased the expression of miR-715, miR-146a, miR-455, miR-322, mir-98, and miR-470 in C2C12 myotubes. The relative expression values from the QRT-PCR analysis were plotted for each gene are mean ± SD (n = 3). The values significantly different from corresponding untreated control (p-value ≤0.01) were represented with ‘*’.

It is estimated that among several thousand human genes, up to one-third of the mRNA, are potential targets for regulation by miRNAs encoded in the genome [40]. To understand the TWEAK-induced regulatory mechanisms that occurs at post-transcriptional level and involves miRs interaction with a target site in the mRNA, we investigated the effect of TWEAK on the expression of various miRs using low density miR array. Out of nearly 760 miRNAs present in our array experiment, about 150 miRs were differentially regulated by TWEAK with p-value≤0.05 and ≥2-fold change. Some of the important miRs with known/putative targets and differentially regulated by TWEAK are presented in Figure 3. Our results showed that TWEAK reduced the expression of muscle-specific miR-1, miR-133a, miR-133b and miR-206 in addition to several other miRs including miR-27, miR-23, miR-93, miR-199, miR-107, and miR-192 (Figure 3A). Moreover, TWEAK also significantly increased the expression of miR-715, miR- 146a, miR-455, miR-322, mir-98, and miR-470 in TWEAK-treated C2C12 myotubes (Figure 3B).

**Figure 4:**
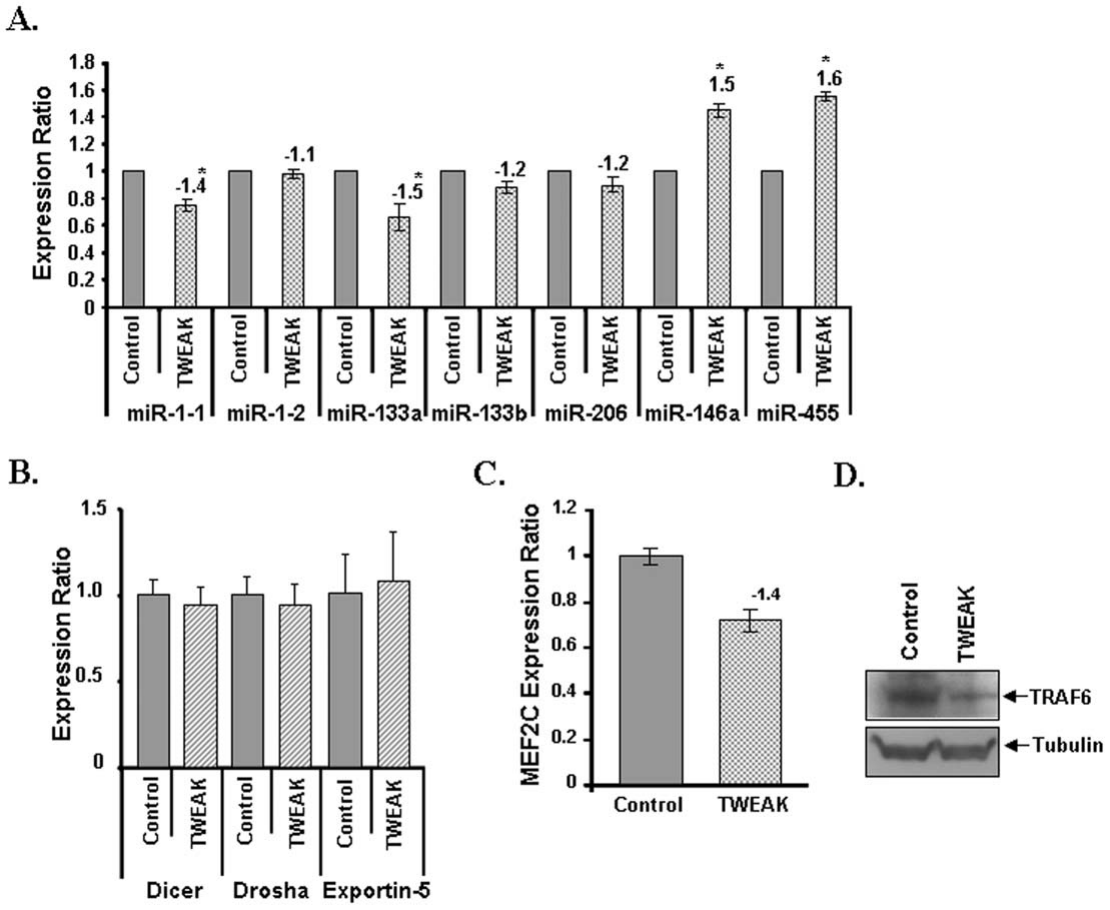
Validation of differentially expressed miRs and their regulatory enzymes by QRT-PCR in TWEAK-treated C2C12 myoblasts. **A).** TaqMan qRT-PCR analysis of miR-1-1, miR-1-2, miR-133a, miR-133b, miR-206, miR-146a, miR-206, miR146a, and miR-455 in TWEAK-treated C2C12 cells. The normalized expression ratios were plotted for each miRNA are mean ± SD (n = 3). The numbers above the bar represents the fold changes with TWEAK treatment against control ‘*’ represents the statistical significance (p-value ≤0.01). **B).** QRT-PCR analysis showed no significant difference in the expression ratio of Dicer, Dorsha and Exportin-5 between control (n = 3) and TWEAK-treated myotubes (n = 3). **C).** TWEAK-treatment significantly reduced the expression of MEF2C transcription factor. The relative expression values were plotted for MEF2C are mean ± SD (n = 3). **D).** C2C12 myotubes were treated with 10 ng/ml of TWEAK for 18h followed by isolation of total protein and performing Western blot. Equal amounts of proteins were loaded on 10% SDS-PAGE gel. Representative immunoblots from two independent experiments presented here showed that TWEAK significantly reduced the protein level of TRAF6 in C2C12 myotubes.

We next investigated whether the expression of some of the miRs found to be altered in response to TWEAK treatment in our array experiment can be validated by independent TaqMan QRT-PCR assays. We studied the expression of miR-1-1, miR-1-2, miR-133a, miR-133b, miR-206, miR-146a, and miR-455. The TaqMan QRT-PCR analysis showed directional correspondence with our low density miRNA-array (Figure 4A). Since our array experiment and QRT-PCR assays were designed to measure the levels of only mature miRs, we also investigated whether the TWEAK regulates the differential expression of these miRNAs at transcriptional level or at post-transcriptional level by measuring the expression levels of their processing enzymes using QRT-PCR assays. Processing of pre-miRs into mature miRs involves a series of reactions that involves regulatory enzymes such as Dicer, Dorsha, and Exportin-5. The altered expression of these enzymes can affect the levels of mature miRs [41]. As shown in Figure 4B, treatment of myotubes with TWEAK for 18h did not affect the transcript levels of Dicer, Dorsha, or Exportin-5 indicating that TWEAK may be affecting the expression of miRs but not their processing from pre-miRs to mature miRs.

Previous studies have suggested that myogenic transcription factors (MRFs) such as MEF2c regulates the expression of various muscle-specific miRs by binding in their promoter/enhancer regions [35], [42], [43]. Indeed, consensus binding sites for MEF2 have been identified in the enhancer and promoter region of miR-1 and miR-133 [42], [43]. By performing QRT-PCR, we validated that the mRNA levels of MEF2C are significantly diminished in TWEAK-treated myotubes (Figure 4C).

**Figure 5:**
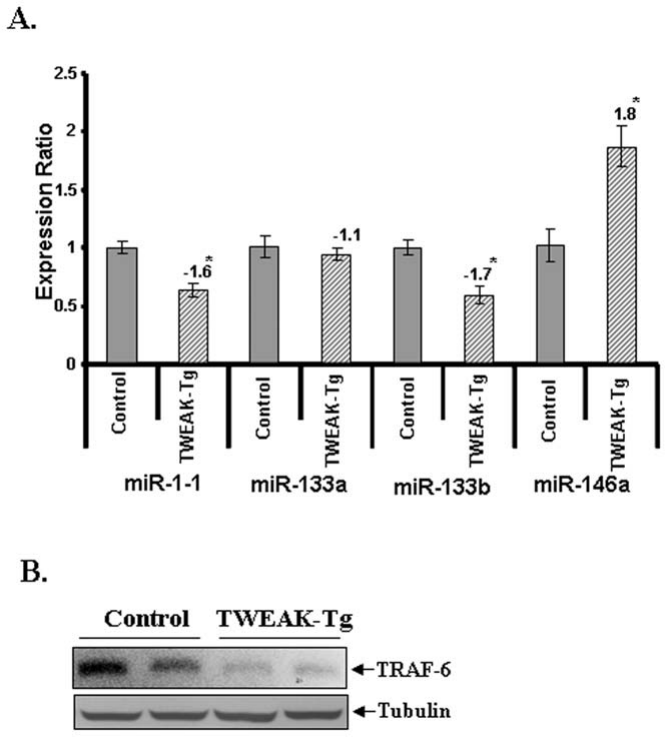
Expression profiles of select microRNAs and TRAF-6 proteins in skeletal muscle of TWEAK-Tg mice. **A)** TaqMan QRT-PCR analysis of miR-1-1, miR-133a, miR-133b, and miR-146a in skeletal muscles of TWEAK-Tg mice. Gastrocnemius muscle from 6 months old TWEAK-Tg mice and littermate control mice were taken and total RNA enriched with small RNAs was isolated for TaqMan qRT-PCR analysis. The normalized expression ratios were plotted for each miRNA are mean ± SD (n = 3). ‘*’ represents the statistical significance (p-value ≤0.01). **B).** Gastrocnemius muscle of 6 months old TWEAK-Tg mice and littermate control mice were taken and total protein was isolated for Western blotting analysis. Representative immunoblot presented here show that the levels of TRAF-6 are considerably reduced in skeletal muscle of TWEAK-Tg (n = 4) mice compared to control (n = 4) mice. Equal amounts of protein loading were ensured by the expression levels of β-actin.

Although TWEAK was found to regulate the expression of various miRNAs, it was not clear whether the altered levels of miRs also affect the expression of their target genes in myotubes. A recent study has suggested that NF-κB transcription factor induces the expression of miR-146a, which is found to be up-regulated in many muscle disorders [34], [44]. To identify the putative targets for this miR, we used miRDB (http://mirdb.org/miRDB/), an online database for miR target prediction and functional annotations in animals. This database uses a bioinformatics tool called MirTarget2, which was developed by analyzing thousands of genes down-regulated by miRs [45]. From this analysis, we found that miR-146a can target TNF receptor-associated factor 6 (TRAF6) with target score more than 95 (Table 3). The role of miR-146a in regulation of TRAF6 levels have also been previously validated by Taganov et al [44]. Moreover, our Western blot experiments confirmed that the protein levels of TRAF6 are reduced in TWEAK-treated C2C12 myotubes (Figure 4D), which is consistent with the increased expression of miR-146a. We also investigated the *in vivo* effects of TWEAK on the expression of various miRs in skeletal muscle. Similar to TWEAK-treated myotubes, the levels of miR1-1 and mir-133b were found to be reduced whereas the levels of miR-146a were increased in skeletal muscle of TWEAK-Tg mice compared to their littermate controls (Figure 5A). The level of miR-133b was also somewhat reduced in TWEAK-Tg mice but it was not significantly different from control mice (Figure 5A). Furthermore, we also found that the levels TRAF6 (a target for miR-146a) were significantly diminished in skeletal muscle of TWEAK-Tg mice compared to control mice (Figure 5B).

**Table 3:** List of differentially expressed miRNAs with known targets/cellular processes.

Detector	Fold	Target/cellular process
**let-7**	−2.35	Hox3a
**let-7e**	−26.467	SOS2, Endoribonuclease Dicer
**miR-107**	−3.1953	Endoribonuclease Dicer, SOS2, decrease proapoptotic signaling, proliferation and remodeling of muscles
**miR-1-1**	−3.2	Increase cell growth, proliferation and remodeling of muscles
**miR-133a**	−3.4278	Increase cell growth, proliferation and remodeling of muscles
**miR-133b**	−2.8	Increase cell growth, proliferation and remodeling of muscles
**miR-146a**	18.6416	Traf6, Delays differentiation through Numb.
**miR-148b**	−2.6183	decrease apoptosis
**miR-152**	−2.3341	Increase cell growth
**miR-17**	−6.2545	Bcl2, N-myc
**miR-181c**	−3.5756	proliferation and remodeling of muscles
**miR-190b**	−2.2	Binds to Ubiquitin-specific protease 46, increase cell growth
**miR-192**	−2.4871	Increase cell growth
**miR-199a-3p**	−1.9	Activin receptor IIA, Map3k4
**miR-218-1**	−2.2887	Increase cell growth
**miR-23b**	−2.1623	Increase Cell growth, proliferation
**miR-26a**	−2.4565	decrease proapoptotic signaling
**miR-27a**	−2.7	Ubiquitin-conjugating enzyme E2N
**miR-27b**	−3	Ubiquitin-conjugating enzyme E2N
**miR-296-3p**	−7.3378	Increase cell growth, decrease apoptosis
**miR-322**	8.7	Hydroxysteroid (17-beta) dehydrogenase 7
**miR-455**	129.249	Up-regulated brown adipocyte differentiation
**miR-470**	3.2	TGFB-induced factor homeobox 1
**miR-715**	18.25	Fucosyltransferase 1
**miR-7a**	−6.2174	Increase cell growth, decrease apoptosis
**miR-93**	−48.423	Map3k14 (NIK)
**miR-98**	1.8	Tripartite motif-containing 6, insulin-like growth factor 2 mRNA binding protein 1

### TWEAK Regulates Distinct Cellular Networks in C2C12 Myotubes

**Figure 6:**
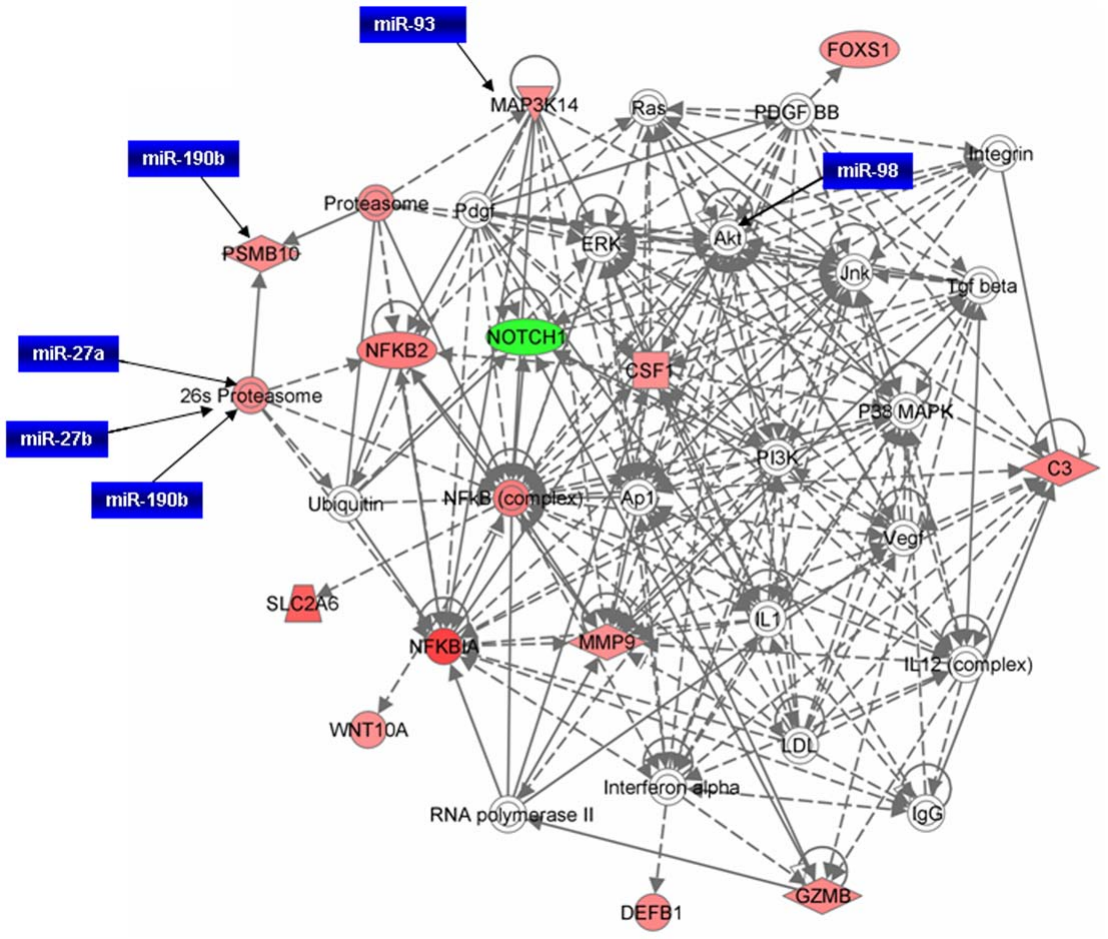
Network of genes up-regulated by TWEAK in microarray experiment. NF-κB and proteasome pathways are major pathways affected by differentially regulated genes by TWEAK. Although many of the microRNAs differentially expressed by TWEAK may not be targeting the differentially regulated genes directly, they can regulate indirectly through other intermediary molecules. For example let-7a and miR-98 may have an indirect effect on expression of Nocth1 by regulating Akt pathway. The solid lines connecting molecules here represent a direct relation and dotted lines an indirect relation. The gene network presented here was adopted from Ingenuity pathway analysis tool with differentially regulated genes by TWEAK with p-values ≤0.05 and ≥1.5-fold. The genes shown in red are up-regulated in microarray data whereas down-regulated genes are shown in green color. Differentially expressed miRNAs (in blue colored boxes) having their putative targets are superimposed on the network.

**Figure 7:**
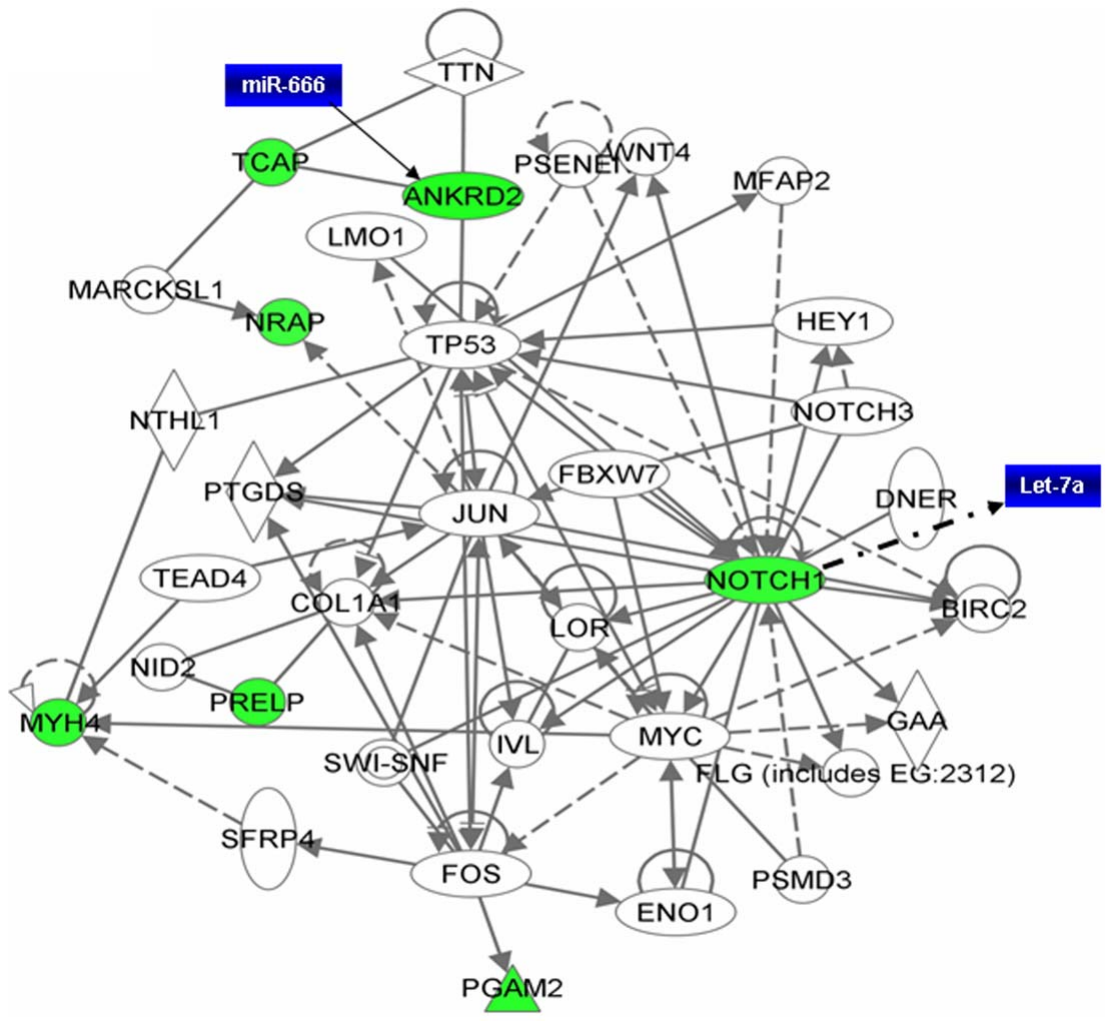
Gene network of down-regulated genes by TWEAK and their possible regulatory miRNAs. Notch1 signaling is major pathway down regulated by TWEAK in C2C12 myotubes. Genes represented in green boxes are those which were found to be significantly down-regulated in our microarray experiment. The genes shown without color are intermediate to the network and are not found in our microarray data. The solid lines connecting molecules here represents a direct relation and dotted lines an indirect relation. This network was obtained from IPA using differentially regulated genes by TWEAK with p-values ≤0.05 and ≥1.5-fold and was superimposed with the miRNAs (blue colored boxes) having their putative targets.

In order to understand the interaction between different genes, we generated common networks using Ingenuity Pathway Analysis (IPA) software. The dataset of differentially expressed genes by TWEAK in C2C12 myotubes with selected stringency (p value≤0.05 and fold ≥1.5) was uploaded into the IPA software tool. Networks of these genes were then algorithmically generated based on their connectivity. The graphical representation of the molecular relationships between genes developed by IPA is presented in Figure 6 and Figure 7. Based on the input information, the genes that are down-regulated are shown in green and the up-regulated genes are shown in red (Figure 6 and 7).

Several transcription factors and protein kinases such as NF-kB2, FoxS1, Notch1, Wnt10A, MMP-9, PSMB10, colony stimulating factor 1 (CSF1), and MAP3K14 were found to be involved in the network related to inflammation, proteolysis, cell survival, proliferation and differentiation (Figure 6). This network also showed that many of these genes are regulated by each other either directly or indirectly. The networks related to cellular development and connective tissue disorder showed that enzymes such as phosphoglycerate 12 mutase (glycolysis), muscle proteins such as myosin heavy chain 4 (Actin cytoskeleton signaling), nebulin-related anchoring protein (Actin binding protein) were significantly down regulated by TWEAK (Figure 7).

Since the targets of many of the miRNAs that are regulated by TWEAK in skeletal muscle are not yet known, we identified the putative target genes and the cellular processes which these micro RNAs affect using miRDB online tool. As shown in Table 3, the microRNAs regulated by TWEAK are involved in regulation of various genes and distinct cellular responses with target score ≥90. Using these miRs, we also generated a network of pathways from differentially regulated genes in cDNA microarray data set. Interestingly, miRNAs network was found to considerably overlap with mRNA networks suggesting that miRNAs may play important roles in the regulation of gene expression in muscle cells (Figures 6 and 7). Collectively, our *in vitro*, *in vivo* and *in silico* experiments show that TWEAK differentially affects the expression of various miRNAs which regulate distinct cellular responses.

## Discussion

### TWEAK Active Multiple Signaling Pathways in Skeletal Muscle Cells

Microarray analysis of TWEAK-treated myotubes has revealed that TWEAK differentially regulates the expression of approximately 25% (p<0.05) of total 25,000 genes probed. However, the number of genes which showed major changes (>1.5 fold) were only in the range of hundred and the functions of some of the differentially regulated genes is not yet known (Table 1). Interestingly, some of the genes affected by TWEAK are also similarly regulated in atrophying muscles in response starvation and unloading [25], [26]. Accumulating evidence suggests that NF-κB is one the most important signaling pathways, activation of which leads to skeletal muscle wastage [1]. The activation of NF-κB can occur through two parallel pathways. The canonical NF-κB signaling pathway involves the upstream activation of IκB kinase-β (IKKβ) and subsequent phosphorylation and degradation of IκB proteins [46], [47], [48]. In contrast, the activation of the alternative NF-κB pathway requires the upstream activation of NF-κB-inducing kinase (NIK or MAP3K14) and IKKα and the proteolytic processing of NFkB2 (p100 subunit) into p52 protein [47], [48]. Our study suggests that TWEAK augments the expression of both NIK (i.e. MAP3K14) and NFkB2 in myotubes (Figure 1B, D, and Table S1). Similarly, increased expression of NIK and NFkB2 were also noticeable in skeletal muscle of TWEAK-Tg mice (Figure 2A and C) further confirming that TWEAK increases the expression of the components of alternative NF-κB signaling pathway. Interestingly, our microarray experiment did not show any increase in the levels of Nfkb1 (e.g. p105 or p50) and RelA, the major components of classical NF-κB pathway [47], [48]. However, we can not articulate that TWEAK does not activate classical NF-κB pathway in skeletal muscle cells. NFkB1 is present in abundance in cytoplasm of the cell and it is rapidly activated in response to various extracellular stimuli through upstream activation of a series of protein kinases [47], [48]. Previously published reports from our group and others have demonstrated that TWEAK increases the activation of both classical and alternative NF-κB pathways [49], [50]. Indeed, the increased expression of Nfkbia (i.e. IκBα), cyclinD1, and MMP-9, which are predominately regulated through classical NF-κB pathway [51] in our microarray and QRT-PCR assays (Figure 1) is suggestive of the fact that TWEAK also augments the activity of classical NF-κB signaling pathway in muscle cells. However, it is important to consider that while TWEAK increases the expression of a number of NF-κB-related genes, all of them may not be involved in TWEAK-induced skeletal muscle-wasting.

Our microarray analysis also revealed that TWEAK inhibits the expression of Notch-1 which was confirmed by performing QRT-PCR and Western blot (Figure 1C and 1D). Similar, reduction in Notch1 levels was observed in skeletal muscle of TWEAK-Tg mice (Figure 2B and 2C). Notch-1 receptors are transmembrane proteins which are expressed in a broad range of tissues and function in diverse developmental and cell maturation processes [52], [53]. Besides its role in regulation of the activity of many other transcription factors, recent studies have shown that constitutively Notch-1 functions as a novel IκB-like molecule and regulates NF-κB-mediated gene expression through a direct interaction with the NFkB1 (i.e. p50) subunit [54], [55]. This interaction prevents NF-κB from binding to NF-κB recognition sites in DNA to regulate NF-κB-dependent gene expression [54], [55]. Therefore, the reduced levels of Notch1 (Figure 1C and 1D) may be responsible, at least in part, for sustained activation of NF-κB in skeletal muscle cells in response to TWEAK. It is also of interest to note that the expression levels of Notch1 are significantly reduced in atrophying skeletal muscle in response to unloading or denervation [56] suggesting that down-regulation of Notch1 may be a common phenomenon in muscle atrophy in response to different stimuli including TWEAK.

In addition to NF-κB, our microarray analysis indicates that TWEAK affects the expression of several genes involved in different cell signaling pathways such as Wnt, MAPK, PI3K/Akt, TGF-β, interferon-γ (IFN-γ), and ubiquitin-proteasome system (Figure 6). While there are published reports demonstrating that TWEAK affects the activation of NF-κB MAPK, PI3K/Akt, and ubiquitin-proteasome systems [17], [20], [21], [22], [49], [50], the present study provides the first evidence regarding a potential role of TWEAK in regulation of Wnt, TGF-β and IFN-γ pathways (Figure 6). The Wnt gene family consists of structurally related genes which encode secretary signaling proteins [57]. These proteins have been implicated in oncogenesis and in several developmental processes, including regulation of cell fate and patterning during embryogenesis [57]. Interestingly, NF-κB is the key pathway for the transactivation of Wnt10a [58], [59]. In a recent study, Brack et al have demonstrated that in aged animals which show significant muscle wasting, the conversion of satellite cells from a myogenic to a fibrogenic lineage occurs due to the activation of the canonical Wnt signaling pathway [60]. It is important to note that the levels of proinflammatory cytokines are increased in skeletal muscle and in circulation during aging and in several chronic diseases [1]. Therefore, it is possible that proinflammatory cytokines such as TWEAK mediates the loss of skeletal muscle mass and causes fibrosis through the activation of Wnt pathway. Indeed, our analysis of differentially regulated genes in TWEAK-treated myotubes by Ingenuity pathway analysis also indicates that TWEAK affects the activation of several toxic pathways including those involved in initiation and manifestation of fibrosis, oxidative stress, and mitochondrial dysfunction (Table 2).

### TWEAK Modulates the Expression of Select Cytoskeleton Molecules in Skeletal Muscle

The interaction between myosin and actin is the molecular basis of muscle contraction and ATP hydrolyzed by myosin is the energy source for mechanical power output. Myosin heavy chain (MyHC) isoforms determine the contractile properties of the myosin molecule and are considered as molecular markers of the fiber type [61]. So far nine myosin isoforms, each coded by a distinct gene, have been found to be expressed in striated muscles and incorporated in the thick filaments [61]. Among them, three fast type MyHC isoforms expressed in adult fast skeletal muscle fibers (called 2A, 2X, and 2B, coded by the genes MYH2, MYH1, and MYH4, respectively) have been found to be the major proteins that undergo proteolytic degradation in various atrophy conditions [1], [11], [62], [63]. Using an antibody that recognizes all the three fast isoforms of MyHC (MyHCf), we have previously reported that TWEAK induces the degradation of MyHCf through the activation of NF-κB and ubiquitin-proteolytic systems [20]. However, there are no reports of TWEAK directly regulating any of these MyHCf isoforms. Our microarray analysis and independent QRT-PCR in this study has suggested that TWEAK may also reduces the expression of MYH4 (i.e. MYHC-2B) in myotubes. The reduced expression of MYH4 also suggests a possibility that TWEAK may inhibit the differentiation and/or dedifferentiation of C2C12 cells. Indeed, we have previously reported that TWEAK inhibits the expression of MyHC and differentiation of C2C12 myoblasts in to myotubes [21].

Interestingly, we have also found that TWEAK down regulates the expression of ankyrin repeat domain 2 (Ankrd2), a structural constituent of striated muscle [64] and Tcap, a 19 kDa sarcomeric protein that is located in the periphery of Z-discs [65]. Mutations in TCap lead to autosomal recessive girdle muscular dystrophy or LGMD type 2 and severe weakness of leg muscle [66], [67]. It has been recently demonstrated that knockdown of TCap in C2C12 myoblasts using siRNA reduces the expression of myogenic regulatory factors MyoD and myogenin suggesting that TCap might be required for the differentiation and maintenance of skeletal muscle mass [68]. Interestingly, we have previously reported that TWEAK reduces the expression of MyoD and myogenin in C2C12 cultures [21]. Since TWEAK significantly reduced the expression of TCap in cultured myotubes within 18h, these data suggest that TWEAK may also be affecting the levels of MyoD and myogenin through inhibiting the expression of TCap. We have also found that the levels of TCap and Ankrd2 are significantly reduced in skeletal muscle of TWEAK-Tg mice (Figure 2B). Furthermore, Ingenuity Pathway Analysis (IPA) showed that Ankrd2 and TCap interact with each other (Figure 7) which is in agreement with a previously published report [69].

In addition to structural proteins, skeletal muscle also expresses many metabolic enzymes required for energy production. The muscle-specific isoform (type M, PGAM2) of phosphoglycerate mutase (PGAM) is a housekeeping enzyme which catalyzes the conversion of 3-phosphoglycerate into 2-phosphoglycerate in the glycolysis process to release energy [70]. PGAM2 is developmentally regulated during myogenesis. Mutations in human have been shown to cause PGAM2 deficiency, which results in acute muscle dysfunction with exercise intolerance and muscle breakdown [46]. Our experiments suggest that TWEAK significantly reduces the expression of PGAM2 not only in cultured myotubes (Figure 1C) but also in skeletal muscle of TWEAK-Tg mice (Figure 2B). Furthermore, our microarray data has confirmed our previous findings [71] that TWEAK augments the expression of extracellular protease matrix metalloproteinase-9 (MMP-9) and reduces the levels of MMP-2 and TIMP2 (Figure 1). Altered expression and production of these molecules in response to TWEAK may cause extracellular matrix abnormalities during muscle-wasting [1], [8], [71].

### TWEAK Regulates the Expression of Several MicroRNAs (miRs) in Skeletal Muscle

The low-density miR array revealed that TWEAK reduces the expression of a large number of miRs which coincidently is in directional correspondence with the up-regulation of majority of genes in our cDNA microarray data with the selected stringent p-values and fold changes (Table 1). Differential expression of relatively fewer miRs when compared to the large number of differentially regulated genes in microarray data also suggests the possibility of targeting more than one gene by each miRNA. Additionally, because miRNAs target genes can directly influence the expression of many other genes indirectly, many of the miRNAs differentially expressed could also be involved in the regulation of some non-target genes [72]. Recently, a few muscle-specific miRs such as miR-1, miR-133a, miR-133b, and miR-206 (also called myomiRs) have been identified which are essential for muscle cell proliferation, differentiation, and maintenance [35]. Expression of miR-1 and miR-133a in embryonic stem cells and other non-muscle cell types showed that they promote the differentiation into the skeletal muscle lineage [35]. Unlike other myomiRs which are also expressed in cardiac tissues, miR-133b and miR-206 are specifically expressed in skeletal muscle though their biological functions are yet to be established. Interestingly, our low density miRs array and independent TaqMan QRT-PCR assays demonstrate that TWEAK reduces the expression levels of miR-1, miR-133a, miR-133b, and miR-206 in skeletal muscle cells (Figure 3A and Figure 4A). Furthermore, the level of at least miR-1 was also found to be significantly reduced in skeletal muscle of TWEAK-Tg mice (Figure 5A). Recent studies have demonstrated that myogenic transcription factors such as serum response factor (SRF), MEF2c, and MyoD control the expression of myomiRs in skeletal and cardiac muscles (reviewed in [35]). We have previously demonstrated that TWEAK reduces the levels of MyoD and myogenin in differentiating C2C12 cultures [21]. Our microarray and QRT-PCR assays in this study have also shown that TWEAK inhibits the expression of MEF2c transcription factor in cultured myotubes (Figure 4C). MEF2C is particularly important for miR-1 and miR-133a and miR-1 further regulates MEF2C levels [42], [43].

In addition to MyomiRs, TWEAK also down-regulated a few more miRNAs such as miR-27a and b, miR-93, miR-199a-3p, miR-107, miR-192, and miR-23b (Fig. 5A). Though miRNAs have been explored extensively in recent years, the targets of many miRNAs are yet to be identified. For this purpose, we have utilized miRNA database (http://mirdb.org/miRDB/) to identify the putative targets of selected miRNAs. From the miRNA database, we identified that miR-27a and b targets ubiquitin-conjugating enzyme E2N with target score above 90. Ubiquitin-conjugating enzyme is an important component of ubiquitin-proteasome pathway, which causes muscle protein degradation in various atrophy conditions [1], [4], [73], [74]. Interestingly, TWEAK has been found to induce the ubiquitination of muscle proteins both in vivo and in vitro [20]. The miRNA database also identified that miR-93 can target MAP3K14 (i.e. NIK) which is involved in NF-κB activation. This suggests that the down-regulation of miR-27a & b and miR-93 leads to activation of ubiquitin-conjugating enzyme leading to up-regulation of ubiquitin-proteasome pathway and NIK in alternative NF-κB signaling pathway thereby inducing muscle atrophy (Figure 6). From the miRNA database, we also observed that TWEAK down-regulates miRNAs that are targeting proliferation and remodeling of muscles (miR-107), matrix metalloprotease such as aggrecanase-2 (miR-192), adamtsl-3 (miR-199-3p), and genes involved in increasing cell growth and proliferation, and microtubule-associated proteins (miR-23b) (Table 3).

The Low-density miRNA arrays of TWEAK-treated C2C12 myotubes also showed upregulation of a few select miRs. Out of the up-regulated miRs, miR-146a and miR-455 have known targets (Table 3). A recent study by Kuang et al showed that miR-146a targets Numb, which promotes satellite cell differentiation towards muscle cells and inhibition of miR-146a by antago-miR146a rescued the expression of Numb and facilitated the differentiation of C2C12 cells [75]. The miRNA database also suggests that miR-146a has a putative target TRAF6 (with target score ≥95). TRAF6 belongs to E3 ubiquitin ligase family which induces the activation of multiple signaling proteins including Akt through formation of Lysine-63-linked poly-ubiquitin chains [76]. The up-regulation of miR-146a in TWEAK-treated C2C12 suggests that one of the potential mechanisms by which TWEAK might be inducing loss of skeletal muscle mass is through down-regulation of Numb and TRAF6. Indeed, our Western blot data suggest that the levels of TRAF6 are reduced in TWEAK-treated myotubes (Figure 4D) and in skeletal muscle of TWEAK-Tg mice (Figure 5B). It is also noteworthy that the expression of miR-146a is regulated through the activation of NF-κB [44], which is activated in response to TWEAK-treatment. Since TRAF6 contributes to the phosphorylation and activation of Akt [76], reduced level of TRAF6 in TWEAK-treated myotubes is consistent with our previous findings that TWEAK inhibits the activation of Akt in C2C12 myotubes [20].

Reduction in skeletal muscle mass and increase of adipocytes (body fat) are the common features of atrophying skeletal muscle [1], [2], [3]. Recent studies have suggested that miR-455 is linked to the up-regulation of brown adipocyte formation. deCastro Rodrigues et al [77] showed the occurrence of fat cell invasion in long-term denervated skeletal muscle. In addition, Eisenberg et al [34] have reported that the levels of miR-455 were increased about two fold in dystrophic muscle of facioscapulohumeral muscular dystrophy, limb girdle muscular dystrophy 2A, and nemaline myopathy. The up-regulation of this miR-455 in our miRNA-array and QRT-PCR assays further signifies its potential role in TWEAK-induced skeletal muscle-wasting (Figure 4A).

### Conclusions

The data presented in this study suggest that TWEAK affects the expression of several genes and related miRs in skeletal muscle cells. These genes and miRs are involved in the regulation of various molecular pathways/processes including ubiquitin-proteasome pathway, extracellular matrix degradation, brown adipocyte formation, and muscle cell proliferation and differentiation. The study has also identified several important genes and miRs that are differentially expressed in skeletal muscle in response to TWEAK. Similar molecules might be involved in skeletal muscle wasting in response to other catabolic stimuli.

## Materials and Methods

### Reagents

Horse serum was purchased from Sigma Chemical Company (St. Louis, MO). Recombinant mouse TWEAK protein and antibodies against MMP-9 and MMP2 were purchased from R&D Systems (Minneapolis, MN). Antibodies against IκBα and Notch1 were purchased from Santa Cruz Biotechnology (San Diego, CA). Tubulin and NFkB2 antibodies were obtained from Cell Signaling Technology (Beverly, MA). TRAF6 antibody was obtained from Millipore (Bedford, MA). Primers for PCR were synthesized by Integrated DNA Technologies (Coralville, IA) or Sigma-Genosys (Woodlands, TX).

### Cell culture

C2C12 myoblastic cell line was obtained from American Type Culture Collection (Rockville, MD). These cells were grown in Dulbecco's modified Eagle's Medium (DMEM) containing 20% fetal bovine serum. C2C12 myoblasts were differentiated into myotubes by incubation in differentiation medium (DM, 2% horse serum in DMEM) for 96h as described [21], [22]. Myotubes were maintained in DM and medium was changed every 48h.

### Animals

Transgenic (Tg) mice expressing TWEAK in skeletal muscle (TWEAK-Tg) have been described previously [20]. Since TWEAK-Tg mice were generated in B6D2F1 background, these mice were crossed with C57BL/6 mice for 7 generations before using for this study. All the experiments with animals were approved by the Institutional Animal Care and Use Committee of the University of Louisville.

### cDNA Microarray

Total RNA was isolated from control and TWEAK-treated C2C12 myotubes using the Agilent total RNA isolation kit (Agilent Technologies, Palo Alto, CA). Any contaminating DNA was removed using DNA-free™ kit from Ambion (Ambion, Austin, TX). The total RNA concentration was determined by NanoDrop spectrophotometer, and RNA quality was determined by 18 S/28 S ribosomal peak intensity on an Agilent Bioanalyzer. Each experiment was performed with a minimum of five replicates. Custom cDNA slides were spotted with Oligator “MEEBO” mouse genome set with 38,467 cDNA probes (Illumina, Inc., San Diego, CA), which allows interrogation of 25,000 genes. A Q-Array2 robot (Genetix) was used for spotting. The array includes positive controls, doped sequences, and random sequences to insure correct gene expression values were obtained from each array. A total of 250 ng RNA was used to synthesize double stranded cDNA using the Low RNA Input Fluorescent Linear Application Kit (Agilent). The microarray slides were scanned using a GSI Lumonics ScanArray 4200A Genepix scanner (Axon). The image intensities were analyzed using the ImaGene 5.6 software (Biodiscovery, Inc., El Segundo, CA). Expression analysis of microarray experiments was performed with GeneSpring 7.1 (Silicon Genetics, Palo Alto, CA) using the raw intensity data generated by the ImaGene software. Local background was subtracted from total signal intensities and was used as intensity measures. The data were normalized using per spot and per chip LOWESS normalization. Data analysis was performed using SAS (SAS Institute, Cary, NC), R and Q value software. The probe sets with absent calls across all samples were removed to reduce the multiple-testing problem. The expression levels were normalized to the chip median and log transformed. Two–way ANOVA tests were carried out to identify differentially expressed genes. For each probe set, the model 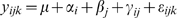 was fit, where 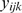 is the log-transformed expression level of the 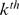 chip in the 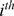 treatment and the 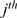 replicate. The variable 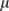 represents the grand mean expression, 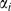 is the effect due to the treatment, 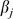 is the effect due to the replicate, 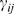 is the interaction effect between treatment and replicate, and 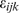 is an error term, which is assumed to be normally distributed with mean 0 and variance 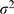. Q values computed using Q value software indicates the false detection rate for each probe set. Ratio comparison was performed by dividing expression levels in TWEAK-treated myotubes with the expression levels in untreated myotubes. Functional classification of select probe sets was performed at NIH DAVID server (http://apps1.niaid.nih.gov/david/upload.asp). Volcano plots were prepared using the R program. The complete raw and normalized microarray data have been submitted in MIAME compliant ArrayExpress (http://www.ebi.ac.uk/microarray-as/ae/) database with accession number E-MEXP-2432.

### MicroRNA (miR) Array Analyses

For miRNA array experiments, total RNA along with small RNAs was isolated from control and 10ng/ml TWEAK-treated C2C12 myotubes using mirVANA miRNA isolation Kit (Ambion, Austin, TX). cDNA was synthesized using Megaplex RT primers (Applied Biosystems, Foster City, CA) which are a set of two predefined pools (pool A and Pool B) of up to 380 stem-looped reverse transcription primers that specifically binds to miRNAs and synthesize cDNA from mature miRNAs. For this we used 500ng of total RNA and 4.5µl of RT reaction mix in a total volume of 7.5µl at the following cycle conditions: 16°C for 2 min, 42°C for 1 min and 50°C for 1 min for total of 40 cycles followed by 85°C for 5 min and bringing the contents to 4°C. The contents were stored at −20°C until further use. The mouse Low Density miRNA array system (Applied Biosystems, Foster City, CA) was used for the miRNA profiling of TWEAK treated C2C12 cells. This miRNA-array kit consists of four plates of plate A and four plates of plate B which contain around 384 miRNAs including four internal controls. For this we used 6µl of cDNA synthesized by using Megaplex RT and 450 µl of TaqMan universal PCR master mix in a total of 900 µl of reaction volume and 100 µl of the reaction mixture was loaded into each port provided in the card (which has 8 ports for each card). Each cDNA was run on both plate A and plate B according to the manufactures protocol. The plates were run in Applied Biosystems Real-time PCR system (7900 HT) by selecting relative quantification (ΔΔCt) and 384 well TaqMan low density array cards. All the samples were run in triplicates. Finally, all the raw data from each plate set was retrieved from the 7900HT machine and was run on RQ manager ver.1.2 (Applied Biosystems, Foster City, CA). The samples were named as Control for control plates and Treatment for TWEAK-treated samples and were checked for their threshold values and peaks. The samples with many peaks or inconsistent peaks were deleted before calculating ΔΔCT and RQ values. The mean values for RQ (which is fold values of treatment compared to control) were used to plot the bar diagrams. The miRNAs with p-value ≤0.05 and fold value of ≥2 were considered for further analysis.

The selected miRNAs were searched for their known targets and those miRNAs with unknown targets were used to identify their putative targets by using miRDB web site (http://mirdb.org/miRDB/) with target score ≥90. The targets/putative targets of selected miRNAs were also analyzed by Ingenuity pathway analysis software to generate interactive pathways and were compared with the pathways obtained from cDNA microarray data.

### Quantitative Real-Time-PCR (QRT-PCR)

The expression of the differentially regulated genes from the microarray data was validated using QRT-PCR using a method as described [8]. The sequence of the primers used is described in Table S2.

The selected miRNAs from the miRNA array were validated using TaqMan QRT-PCR analysis by using their specific primers from Applied Biosystems (Foster City, CA). For this the cDNA for each selected miRNA was synthesized by using the miRNA specific primers supplied by the manufacturer (Applied Biosystems). Briefly, 100 ng of total RNA was taken for cDNA synthesis of each miRNA in a final volume of 20µl by using the miRNA specific primers (Part number 4427975; assay IDs, 002455, 000468, 002222, 002247, 000510, 001637, and 002882) to ensure cDNA synthesis of mature miRNAs as given in the manufactures protocol. U6 was used as an internal control for miRNA in TaqMan QRT-PCR.

### Pathways and Networks Analyses

Relative levels of gene expression were first computed with GeneSpring 7.1 to obtain data sets of differentially regulated genes based on cut-off values of 5% error rate (p<0.05, determined by t-test with Benjamini and Hochberg Multiple Testing Correction). These data sets included up and down regulated genes when C2C12 myotubes were treated with TWEAK. The second step of analysis consisted of identifying canonical pathways. Tab separated (txt) files containing Accession IDs and symbols derived from MEEBO genome set and the normalized expression ratios were then uploaded to Ingenuity Pathways Analysis. Ingenuity Pathways Analysis is a web-delivered bioinformatics tool (IPA 5.0, http://www.ingenuity.com) to identify pathways and functional networks. IPA knowledge database is generated from the peer-reviewed scientific publications that enables discovery. The Accession IDs and symbols in each data set were queried against all genes stored in the IPA knowledge database for pathway analysis. Canonical pathways analysis identified the pathways from IPA library of canonical pathways that were most significant to the data set. The significance of the association between the data set and the canonical pathways was measured in 2 ways: 1) A ratio of the number of genes from the data set that map to the pathway divided by the total number of genes that map to the canonical pathway is displayed. 2) Fisher's exact test was used to calculate a p-value determining the probability that the association between the genes in the data set and the canonical pathway is explained by chance alone.

### Western Blot

Immunoblotting was performed to measure the levels of various proteins in C2C12 myotubes or skeletal muscle tissues of TWEAK-Tg mice using a protocol as described [78].

### Statistical Analysis

Methods used for statistical analysis of the cDNA microarray and microRNA arrays data has been described above in their respective sections. For all other studies, results were expressed as mean ± SD. The Student's *t* test was used to compare quantitative data populations with normal distributions and equal variance. A value of *P *<0.05 was considered statistically significant unless otherwise specified.

## Supporting Information

Table S1 Extreme 50 known genes that are up-regulated or down-regulated by TWEAK in microarray experiment.
(0.12 MB DOC)

Table S2 Sequence of the primers used for quantitative real-time PCR assay.
(0.04 MB DOC)
