# Supplementary material for: Genomic Profiling of Messenger RNAs and MicroRNAs Reveals Potential Mechanisms of TWEAK-Induced Skeletal Muscle Wasting in Mice
Source: PLoS One. 2010 Jan 19;5(1):e8760. doi: 10.1371/journal.pone.0008760 (PMC2808241; doi:10.1371/journal.pone.0008760)
Supplement: Table S1 — Extreme 50 known genes that are up-regulated or down-regulated by TWEAK in microarray experiment. (0.12 MB DOC) [file pone.0008760.s001.doc]

**Table S1.** Extreme 50 known genes that are up-regulated or down-regulated by TWEAK in microarray experiment.

| **Gene Symbol** | **Normalized ratio of Up-regulated Genes** | **Gene Description** | **Gene Symbol** | **Normalized ratio of Down regulated Genes** | **Gene Description** |
| --- | --- | --- | --- | --- | --- |
| Taf2 | 2.761 | TATA box binding protein (TBP)-associated factor | Ankrd2 | 0.572 | ankyrin repeat domain 2 (stretch responsive muscle) |
| Nfkbia | 2.54 | nuclear factor of kappa light chain gene enhancer in B-cells inhibitor, alpha | Ankrd23 | 0.746 | ankyrin repeat domain 23 |
| Slc2a6 | 2.142 | solute carrier family 2 (facilitated glucose transporter), member 6 | Arhgap21 | 0.738 | Rho GTPase activating protein 21 |
| Dlgap2 | 1.865 | discs, large (Drosophila) homolog-associated protein 2 | Armcx2 | 0.742 | armadillo repeat containing, X-linked 2 |
| Polr3k | 1.798 | polymerase (RNA) III (DNA directed) polypeptide K | Asb2 | 0.688 | ankyrin repeat and SOCS box-containing protein 2 |
| Nfkb2 | 1.762 | nuclear factor of kappa light polypeptide gene enhancer in B-cells 2 | Atp2a2 | 0.738 | ATPase, Ca++ transporting, cardiac muscle, slow twitch 2 |
| X66118 | 1.742 | M.musculus mRNA for glutamate receptor subunit GluR5-2c | Cacna1s | 0.746 | calcium channel, voltage-dependent, L type, alpha 1S subunit |
| Mt2 | 1.701 | metallothionein 2 | Clcn7 | 0.752 | chloride channel 7 |
| H2-K1 | 1.681 | histocompatibility 2, K1 | Col4a2 | 0.697 | procollagen, type IV, alpha 2 |
| Krtap16-2 | 1.673 | keratin associated protein 16-2 | Col5a1 | 0.735 | procollagen, type V, alpha 1 |
| Slc9a3 | 1.609 | solute carrier family 9 (sodium/hydrogen exchanger), member 3 | Crip1 | 0.743 | cysteine-rich protein 1 (intestinal) |
| Gzmb | 1.608 | granzyme B | Crym | 0.758 | crystallin, mu |
| Hsd17b9 | 1.605 | hydroxysteroid (17-beta) dehydrogenase 9 | Csrp3 | 0.752 | cysteine and glycine-rich protein 3 |
| Defb1 | 1.602 | defensin beta 1 | D0H4S114 | 0.715 | DNA segment, human D4S114 |
| V1rd18 | 1.602 | vomeronasal 1 receptor, D18 | D14Ertd226e | 0.744 | tetraspanin 14 |
| Cstb | 1.581 | cystatin B | Depdc6 | 0.65 | DEP domain containing 6 |
| Krt1-14 | 1.579 | keratin complex 1, acidic, gene 14 | Fgfrl1 | 0.746 | fibroblast growth factor receptor-like 1 |
| Olfr186 | 1.577 | olfactory receptor 186 | Fst | 0.738 | follistatin |
| Zfp9 | 1.553 | zinc finger protein 9 | Golgb1 | 0.752 |  |
| Pnn | 1.549 | pinin | Gpr151 | 0.731 | G protein-coupled receptor 151 |
| Lxn | 1.547 | latexin | Gpr56 | 0.735 | G protein-coupled receptor 56 |
| Nmyc1 | 1.545 | neuroblastoma myc-related oncogene 1 | Gpx3 | 0.715 | glutathione peroxidase 3 |
| Olfr1392 | 1.544 | olfactory receptor 1392 | Grcc9 | 0.753 | splA/ryanodine receptor domain and SOCS box containing 2 |
| Psmb10 | 1.536 | proteasome (prosome, macropain) subunit, beta type 10 | H2afv | 0.689 | H2A histone family, member V |
| Cd200r4 | 1.531 | Cd200 receptor 4 | Hexa | 0.724 | hexosaminidase A |
| V1rd21 | 1.526 | vomeronasal 1 receptor, D21 | Hey1 | 0.684 | hairy/enhancer-of-split related with YRPW motif 1 |
| Mmp9 | 1.509 | matrix metalloproteinase 9 | Htr6 | 0.741 | 5-hydroxytryptamine (serotonin) receptor 6 |
| Map3k14 | 1.503 | mitogen-activated protein kinase kinase kinase 14 | Idb3 | 0.645 | inhibitor of DNA binding 3 |
| Fkhl18 | 1.502 | forkhead-like 18 (Drosophila) | Ly6a | 0.73 | lymphocyte antigen 6 complex, locus A |
| Isyna1 | 1.501 | myo-inositol 1-phosphate synthase A1 | Maged2 | 0.758 | melanoma antigen, family D, 2 |
| Csf1 | 1.499 | colony stimulating factor 1 (macrophage) | Mb | 0.738 | myoglobin |
| Wnt10a | 1.496 | wingless related MMTV integration site 10a | Mef2c | 0.747 | myocyte enhancer factor 2C |
| Wdr19 | 1.494 | WD repeat domain 19 | Mest | 0.714 | mesoderm specific transcript |
| Olfr552 | 1.492 | olfactory receptor 552 | Myh4 | 0.611 | Mus musculus myosin, heavy polypeptide 4 |
| Cd34 | 1.477 | CD34 antigen | Ndfip1 | 0.751 |  |
| Havcr1 | 1.476 | hepatitis A virus cellular receptor 1 | Notch1 | 0.627 | Notch gene homolog 1 (Drosophila) |
| Olfr395 | 1.472 | olfactory receptor 395 | Nrap | 0.66 | nebulin-related anchoring protein |
| Gm1012 | 1.471 | gene model 1012, (NCBI) | Olfr297 | 0.659 | olfactory receptor 297 |
| Cxcl1 | 1.468 | chemokine (C-X-C motif) ligand 1 | Pald | 0.753 | cDNA sequence X99384 |
| IGKV13-82 | 1.468 | Mus musculus IgVk gq33 pseudogene. | Pgam2 | 0.602 | phosphoglycerate mutase 2 |
| IGKV3-12 | 1.468 | Mouse Ig germline kappa V-region 9.5kb-V-kappa | Prelp | 0.618 | proline arginine-rich end leucine-rich repeat |
| Olfr799 | 1.467 | olfactory receptor 799 | Ptgis | 0.753 | prostaglandin I2 (prostacyclin) synthase |
| Ccnd1 | 1.467 | cyclin D1 | Purb | 0.714 | purine rich element binding protein B |
| Ces2 | 1.463 | carboxylesterase 2 | Sparc | 0.756 | secreted acidic cysteine rich glycoprotein |
| Lzts2 | 1.461 | leucine zipper, putative tumor suppressor 2 | Spon2 | 0.668 | spondin 2, extracellular matrix protein |
| Pramel5 | 1.45 | preferentially expressed antigen in melanoma like 5 | Stx5a | 0.694 | syntaxin 5A |
| Gp38 | 1.447 | podoplanin | Tcap | 0.633 | titin-cap |
| Rkhd2 | 1.443 | Mus musculus ring finger and KH domain containing 2 (Rkhd2) | Tcf4 | 0.679 | transcription factor 4 |
| Optc | 1.441 | opticin | Tgfb3 | 0.732 | transforming growth factor, beta 3 |
| Pigw | 1.437 | phosphatidylinositol glycan anchor biosynthesis, class W | Timp2 | 0.719 | tissue inhibitor of metalloproteinase 2 |
