# Supplementary material for: Genomic Profiling of Messenger RNAs and MicroRNAs Reveals Potential Mechanisms of TWEAK-Induced Skeletal Muscle Wasting in Mice
Source: PLoS One. 2010 Jan 19;5(1):e8760. doi: 10.1371/journal.pone.0008760 (PMC2808241; doi:10.1371/journal.pone.0008760)
Supplement: Table S2 — Sequence of the primers used for quantitative real-time PCR assay. (0.04 MB DOC) [file pone.0008760.s002.doc]

**Table S2.** Sequence of the primers used for quantitative real-time PCR (QRT-PCR) assay.

| **Gene name** | **Forward primer sequence** | **Reverse primer sequence** |
| --- | --- | --- |
| Notch1 | 5'-TTCCTTCTACTGCGAATGTCCG-3' | 5'-AGATGGCTTTGCCGTTGACA-3' |
| NFkB1 | 5'-GAGGAGTACGAGCAAATGGTGAAG-3' | 5'-ATTGCCAAGTGCAGGAACGA-3' |
| NFkB2 | 5'-TAAAGGCTGGTGCTGACATCCA-3' | 5'-GGTGTTCTGAGCAGCATTTAGCAG-3' |
| Psmb10 | 5'-GATTTGAACGGACCTCAGCTCTAC-3' | 5'-AGCGTCATGTTTGGCTGGAA-3' |
| MAP3K14 | 5'-CCAAAGCCAAGGAGAAGACACA-3' | 5'-TTGGCTGTGCCTTTGGTGAT-3' |
| Tcap | 5'-AGGACGTGGCTGAGATCACAAA-3' | 5'-AACTACAAAGCAGCCACTGCCA-3' |
| MyHC4 | 5'-GGAACAGTATGAAGAGGAGCAGGA-3' | 5'-ATTCTGAAGTCGCTGCTTCGTC-3' |
| PGAM2 | 5'-CAAGAGAACCGTTTCTGTGGCT-3' | 5'-CCCACATTTGGTCCGTAACATC-3' |
| Ankrd2 | 5'-GACACCAACGTGAGAGACAAGCTA-3' | 5'-TCCAAGCCCAGGGAAAGAAA-3' |
| CyclinD1 | 5′-CGGATGAGAACAAGCAGACCAT-3′ | 5′-CTGGAAAGAAAGTGCGTTGTGC-3′ |
| MMP2 | 5′-ACAGCCAGAGACCTCAGGGT-3′ | 5′-CAGCACAGGACGCAGAGAAC-3′ |
| MMP9 | 5′-GCGTGTCTGGAGATTCGACTTG-3′ | 5'-CATGGTCCACCTTGTTCACCTC-3' |
| TIMP2 | 5′-GTGACTTCATTGTGCCCTGGG-3′ | 5′-TGGGACAGCGAGTGATCTTGC-3′ |
| Dicer1 | 5'-TGCTCGAGATGGAACCAGA-3' | 5'-TCAGCTGTTAGGAACCTGA GGC-3' |
| Dorsha | 5'-GGATAGGCTGTGGGAAAGGA-3' | 5'-CTTCTTGATGTCTTCAG CCTCC-3' |
| Exportin-5 | 5'-CCACTTCAAACGTCTAATCGCT-3' | 5'-GCCGGAGAAGGAT GCC-3' |
| Rpl26 | 5'-CGAGTCCAGCGAGAGAAGG-3' | 5'-GCAGTCTTTAATGAAAGCC GTG-3' |
| β-Actin | 5'-CAGGCATTGCTGACAGGATG-3' | 5'-TGCTGATCCACATCTGCTGG-3' |
